# *SlTPL1* Silencing Induces Facultative Parthenocarpy in Tomato

**DOI:** 10.3389/fpls.2021.672232

**Published:** 2021-05-20

**Authors:** Mi He, Shiwei Song, Xiaoyang Zhu, Yuxiang Lin, Zanlin Pan, Lin Chen, Da Chen, Guojian Hu, Baowen Huang, Mengyi Chen, Caiyu Wu, Riyuan Chen, Mondher Bouzayen, Mohammed Zouine, Yanwei Hao

**Affiliations:** ^1^Key Laboratory of Horticultural Crop Biology and Germplasm Innovation in South China, Ministry of Agriculture, College of Horticulture, South China Agricultural University, Guangzhou, China; ^2^Laboratory Genomics and Biotechnology of Fruits, INRA, Toulouse INP, University of Toulouse, Toulouse, France; ^3^Institute of Bioengineering, Guangdong Academy of Sciences, Guangzhou, China

**Keywords:** fruit set, cytokinins, tomato, *SlTPL1*, facultative parthenocarpy

## Abstract

Facultative parthenocarpy is of great practical value. However, the molecular mechanism underlying facultative parthenocarpy remains elusive. Transcriptional co-repressors (TPL) act as a central regulatory hub controlling all nine phytohormone pathways. Previously, we proved that SlTPLs participate in the auxin signaling pathway by interacting with auxin/indole acetic acid (Aux/IAAs) in tomato; however, their function in fruit development has not been studied. In addition to their high expression levels during flower development, the interaction between SlTPL1 and SlIAA9 stimulated the investigation of its functional significance via RNA interference (RNAi) technology, whereby the translation of a protein is prevented by selective degradation of its encoded mRNA. Down-regulation of SlTPL1 resulted in facultative parthenocarpy. Plants of SlTPL1-RNAi transgenic lines produced similar fruits which did not show any pleiotropic effects under normal conditions. However, they produced seedless fruits upon emasculation and under heat stress conditions. Furthermore, SlTPL1-RNAi flower buds contained higher levels of cytokinins and lower levels of abscisic acid. To reveal how SlTPL1 regulates facultative parthenocarpy, RNA-seq was performed to identify genes regulated by SlTPL1 in ovaries before and after fruit set. The results showed that down-regulation of SlTPL1 resulted in reduced expression levels of cytokinin metabolism-related genes, and all transcription factors such as MYB, CDF, and ERFs. Conversely, down-regulation of SlTPL1 induced the expression of genes related to cell wall and cytoskeleton organization. These data provide novel insights into the molecular mechanism of facultative tomato parthenocarpy and identify SlTPL1 as a key factor regulating these processes.

## Introduction

The great global production and consumption (Klap et al., 2017; Quinet et al., 2019) of tomato unquestionably make it the most important vegetable crop in the world. In addition to its economic and nutritional importance, the availability of the entire tomato genome, genetic and physical maps, and molecular markers make tomatoes an ideal model plant for the study of fleshy fruit development (Tomato Genome Consortium, 2012; Karlova et al., 2014; Suresh et al., 2014; Zhao et al., 2019). The fruit originates from the development of the ovary, and the initiation of such development is known as fruit set, which comprises the transition from an ovary into a fruit (Wang et al., 2009; Quinet et al., 2019). Low fruit-set rates will reduce fruit production and quality, resulting in great economic losses (McAtee et al., 2013).

In normal fruit development, the onset of fruit set depends on the successful completion of pollination and fertilization (Weterings and Russell, 2004). Increased auxin, cytokinin (CK), and gibberellin (GA) levels in fertilized ovaries promote fruit growth through cell division and subsequent cell expansion (Ozga et al., 2002; de Jong et al., 2009a; Ding et al., 2013). However, when plants undergo unfavorable conditions, such as extreme temperature (heat or cold) or humidity, fruit set will be inhibited because of low pollen viability, which affects microsporogenesis and pollination (Picken, 1984; Sato et al., 2006; Mesihovic et al., 2016). Hence, parthenocarpy, which is the formation of seedless fruit from the ovary in the absence of pollination and fertilization, has been recognized as an important trait to counter harsh environmental conditions (Gorguet et al., 2005). Moreover, consumers prefer seedless over seeded fruits because of their improved fruit quality with high total soluble solids (TSS) content and the separation of seeds from processed products (Ficcadenti et al., 1999; Carmi et al., 2003). There are two kinds of parthenocarpy, namely, obligate and facultative parthenocarpy. The difference is that the former always produces seedless fruits, whereas the latter results in seedless fruits only when pollination is prevented (Mazzucato et al., 1999; Varoquaux et al., 2000). As obligate parthenocarpic fruits are commonly propagated from seeds, only facultative parthenocarpic fruits are of practical value (Klap et al., 2017). The most extensively characterized sources of facultative parthenocarpy in tomato to date are the following mutations: *pat/pat2/pat3/4* (Mazzucato et al., 1998; Fos et al., 2000; Fosa et al., 2001; Pascual et al., 2009); *procera* (Bassel et al., 2008); *entire* (Mazzucato et al., 2015); *auscia* (Molesini et al., 2009); *alq* (Ribelles et al., 2019); *hws* (Damayanti et al., 2019); and *Slagl6* (Klap et al., 2017). However, despite the importance of the trait, the use of these parthenocarpic mutants in breeding programs remains limited.

Parthenocarpic fruit-set can be attained by natural genetic manipulation (Ficcadenti et al., 1999; Carmi et al., 2003), induction via exogenous application of phytohormones (auxins, CKs, and GAs) to the ovary (de Jong et al., 2009a; Ding et al., 2013), elevating the levels of these endogenous phytohormones, or enhancing the response of ovaries and ovules to auxins and GAs (de Jong et al., 2009a; Wang et al., 2009; Takisawa et al., 2018; Matsuo et al., 2020; Wang et al., 2020). At the molecular level, GA is the key hormone in the fruit-set process. Auxin and GA interact and form a feedback loop to promote fruit set in tomato (de Jong et al., 2009a; Hu et al., 2018). Auxin is induced in the fertilized ovules and then transported to the pericarp to activate GA synthesis, which releases ovary growth repression (Serrani et al., 2010). Meanwhile, high GA levels in the gynoecium are essential for the initiation of auxin biosynthesis in the ovules (Vivian-Smith and Koltunow, 1999).

In addition to auxin, ethylene prevents fruit set by inhibiting the perception of GAs (Shinozaki et al., 2015). Furthermore, abscisic acid (ABA) counteracts the effect of GAs on fruit set in pea (Carboneil and Garda-Martinez, 1980). In turn, CK is believed to interact with auxin to promote cell proliferation during fruit development (Srivastava and Handa, 2005; Ding et al., 2013). Unlike auxins, GAs, and CKs, which increase in association with fruit set, ABA and ethylene levels decrease (Kojima et al., 1993; Shinozaki et al., 2015). Recently, ABA was shown to have a negative effect on fruit set. Overexpression of *SlNCED3* increases ABA level in the ovary and reduces fruit-set rate (Kai et al., 2019).

Although many studies have been conducted to elucidate the molecular mechanism responsible for the regulation of obligate parthenocarpy, the regulation of facultative parthenocarpy remains unexplained. Previously, we isolated the TOPLESS gene family from tomato and proposed that genes in this family may participate in the auxin-signaling pathway by interacting with Aux/IAA members in tomato (Hao et al., 2014). However, to date, their function in fruit development has not been studied. SlTPL1, which was expressed to a high level in flowers, interacted with IAA9, whose mutation resulted in facultative parthenocarpic fruit formation (Wang et al., 2009; Mazzucato et al., 2015). As the precise role of SlTPL1 in facultative parthenocarpy remains unclear, in this study, we conducted experiments in which SlTPL1-silenced tomato plants were generated using RNA interference (RNAi) technology. SlTPL1-RNAi plants produced fruit with similar phenotypes that did not show pleiotropic effects under normal conditions. However, they produced seedless fruit upon emasculation and under heat-stress conditions. SlTPL1-RNAi flower buds contained higher levels of CK and lower levels of ABA, while the down-regulation of SlTPL1 resulted in reduced expression levels of CK metabolic genes, thereby inducing the expression of genes related to cell wall and cytoskeleton organization. Based on these data, we propose that SlTPL1 participates in the regulation of facultative parthenocarpic fruit formation by modulating the CK level during fruit development.

## Materials and Methods

### Plant Materials, Growth Conditions, and Plant Transformation

SlTPL1-RNAi transgenic tomato plants were generated by Agrobacterium-mediated transformation according to the method described by Hao et al. (2015). The SlTPL1-RNAi vector was constructed by cloning a specific cDNA fragment (277 bp) of *SlTPL1* into the pHellsgate 12 vector. The primers used in the fragment amplication is TPL1-RNAi-attb1 and TPL1-RNAi-attb2 which were listed in the Supplementary Table 10. Wildtype (*Solanum lycopersicum* L. “Micro-Tom”) and transgenic tomato plants were cultivated in a greenhouse at the College of Horticulture of the South China Agriculture University. The culture medium and growth conditions were as previously described (Guan et al., 2018). The primers used for cloning and verification of transgenic plants are listed in Supplementary Table 10. Flower buds at 2, 4, 6, and 8 mm in length, and at anthesis were sampled for analysis. Petals, sepals, stamens, and carpels were sampled at anthesis. Ovaries at 2 days BA, at anthesis (An), and 4 days post-anthesis stage were also collected for the gene expression study. All tissue samples were immediately frozen with liquid nitrogen and stored at -80°C until use.

### Flower and Fruit Phenotypes

Ten plants from each non-transgenic and transgenic line were used. Twenty flowers were retained per plant and the number of set fruits per plant was recorded to calculate average fruit setting rate. All non-transgenic and transgenic plants were dated at anthesis and fruit breaking stages, and the length of fruit development was calculated. Twenty-five fruits at the breaking plus 7-day stage were used to calculate fruit weight, fruit size, seed number, hue angle value, and TSS content. Fruit at mature green (MG), breaking (Br), Br + 1, Br + 2, Br + 4, and Br + 7 day stages were collected from WT and transgenic plants for ethylene production measurement. At least five fruit at each developmental stage were sampled. A Student’s *t*-test was used to perform statistical analysis. Differences were considered significant at *P* < 0.05.

### Phytohormones Contents Measurement

Ovaries at 2 days before anthesis stage (E0) were collected from WT and SlTPL1 RNAi plant and immediately frozen in liquid nitrogen. Three replicates were prepared for each genotype. Phytohormones contents were detected by MetWare^1^ based on the AB Sciex QTRAP 6500 LC-MS/MS platform. For GA extraction, the internal standards were added to plant samples, and then the fresh plant materials were ground into powder under liquid nitrogen and extracted with 500 ul acetonitrile (ACN) (Darmstadt, Germany). For other hormones extraction, fresh tissues powder were extracted with 1mL methanol/water/formic acid (15:4:1, V/V/V). The sample extracts were analyzed using an LC-ESI-MS/MS system (UHPLC, ExionLC^TM^ AD^2^; MS, Applied Biosystems 6500 Triple Quadrupole). AB 6500 + QTRAP LC-MS/MS system, equipped with an ESI Turbo Ion-Spray interface, operating in both positive and controlled by Analyst 1.6 software (AB Sciex).

### Emasculation and High Temperature Stress

Emasculation treatment was performed on five non-transgenic and five transgenic plants. Stamens were removed from the flower bud 2 days before anthesis and kept in the growth chamber for the fruit-set calculation. Each plant retained 20 emasculated flowers. Five non-transgenic and five transgenic plants were placed in the growth incubator at the beginning of the bud stage; at this point, daytime (16 h) and nighttime (8 h) temperatures were set at 35 and 30°C, respectively, for heat-stress treatment.

### RNA-Seq Analysis

Flower buds of WT and SlTPL1-RNAi line 1 were emasculated 2 days before anthesis. The ovaries of the emasculated flowers from these two genotypes were removed at 0 and at 7 days after emasculation, immediately frozen in liquid nitrogen, and stored at −80°C. Three biological repeats were prepared for each sample, and each sample included at least 20 ovaries. All samples (three biological replicates) were sent to Guangzhou Gene *Denovo* Biological Technology Co., Ltd. (Guangzhou, China) for RNA isolation and RNA-Seq library preparation and sequencing. The cDNA libraries were sequenced using the Illumina HiSeqTM 2500. Sequence read mapping and assembly were as previously described by Song et al. (2018). DEGs were determined using an FDR < 0.05 threshold and an absolute value of | log2 (fold change)| > 1. GSEA was performed on the Guangzhou Gene *Denovo* Biological Technology Co., Ltd. (Guangzhou, China), OmicShare Tools^3^, a free online platform developed by Guangzhou GENE *DENOVO* Biotech.

### RNA Isolation and qRT-PCR Analysis

Total RNA was provided by Gene *Denovo* Biological Technology Co., Ltd. (Guangzhou, China). cDNA was produced using the PrimeScriptTM RT Reagent Kit with gDNA Eraser (TaKaRa, RR047A). qPCR was performed using the LightCycler 480 Real-Time PCR system (Roche, Basel, Switzerland) with SYBR Premix Ex Taq (TaKaRa Bio, Inc.), and relative gene expression was calculated using the expression levels of the housekeeping gene SlUBQ and the 2^–ΔΔCt^ method (Livak and Schmittgen, 2001). The expression of 13 genes selected from RNA-seq was validated by qRT-PCR.

## Results

### *SlTPL1* Expression Level Increased After Fruit Set

**FIGURE 1:**
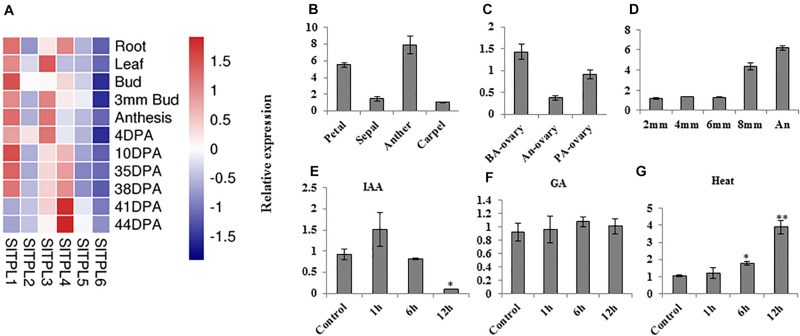
Expression pattern analysis of *SlTPL1* by qRT-PCR. **(A)** Heat map of expression levels of *SlTPL*s family genes in various tomato organs based on the RNA-seq data on the tomexpress website. **(B–D)** Expression pattern of *SlTPL1* in tomato organs by qRT-PCR. **(E–G)** Expression pattern of *SlTPL1* under auxin, GA, and heat treatment. *T*-test was used for statistical analyses of comparison between the control and each treatment, *P < 0.05, **P < 0.01. BA-ovary: ovaries at 2 days before anthesis. An-ovary: ovaries at anthesis. PA-ovary: ovaries at 4 days post anthesis. 2 mm: anther from a 2-mm flower bud; 4 mm: anther from a 4-mm flower bud; 6 mm: anther from a 6-mm flower bud; 8 mm: anther from an 8-mm flower bud; An: anther of a flower at anthesis.

Among all the genes in the *SlTPL* gene family, *SlTPL1* was highly expressed in all tomato organs during flower development (Figure 1A). Using qRT-PCR, we checked the expression level of *SlTPL1* during the process of tomato fruit-set in all flower organs and at different anther developmental stages to obtain a more precise characterization of the expression pattern of *SlTPL1* during flower development. The results showed that *SlTPL1* displayed a reverse trend to that of *SlARF7*, which decreased sharply in the ovary at anthesis and then increased when fruit set was completed (Figure 1C). *SlTPL1* transcripts accumulated in sepals, petals, anthers, and carpels at anthesis, particularly in the anthers (Figure 1B). Furthermore, *SlTPL1* expression increased with anthers development (Figure 1D). Additionally, as auxin and GAs are the main hormones involved in the fruit-set process, we checked the responsiveness of *SlTPL1* to auxin and GA treatment. The results revealed that *SlTPL1* was down-regulated by auxin treatment (Figure 1E) but did not respond to GA treatment (Figure 1F).

### *SlTPL1* Silencing Increased Tomato Fruit-Set Capacity After Flower Emasculation and Under Heat-Stress Conditions

**FIGURE 2:**
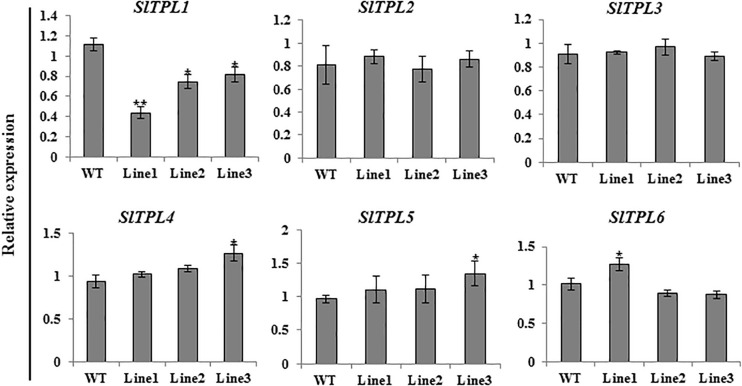
Expression level analysis of six *SlTPL*s (*SlTPL1, SlTPL2, SlTPL3, SlTPL4, SlTPL5*, and *SlTPL6*) in WT and SlTPL1-RNAi lines by qRT-PCR. *T*-test was used for statistical analyses of comparison between the WT and each line, *P < 0.05; **P < 0.01.

**FIGURE 3:**
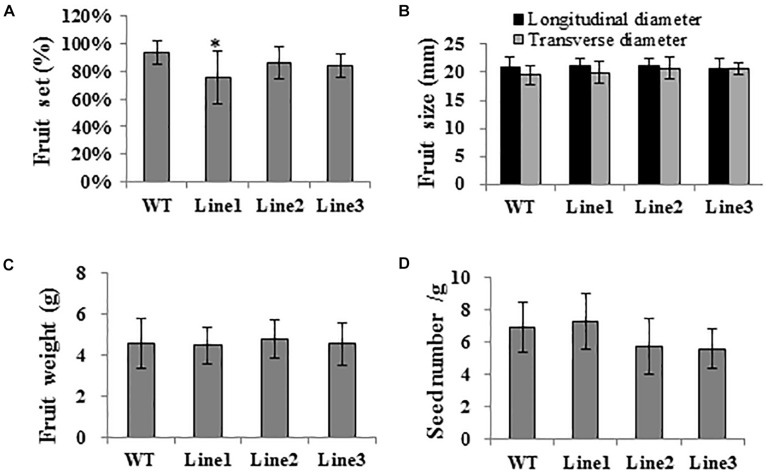
**(A-D)** Phenotyping of SlTPL1-RNAi tomato plants. Characterization of fruit set, fruit size, fruit weight, and seed number in SlTPL1-RNAi plants. *T*-test was used for statistical analyses of comparison between the WT and each line, *P < 0.05.

Aiming to elucidate the role of SlTPL1 in the determination of fruit-set capacity in tomato, we used RNAi technology to develop *SlTPL1* down-regulated transgenic plants. qRT-PCR was performed to check the level of expression of *SlTPL1* in these homozygous transgenic plants. The results showed that *SlTPL1* was significantly reduced in three RNAi lines (line1, line2, and line3) compared with that in the non-transgenic lines (Figure 2). Two of the three SlTPL1 RNAi lines (line2, line 3) retained 70% of the control mRNA level; one SlTPL1 RNAi line (line1) showed the greatest decrease in *SlTPL1* expression, retaining only 43% of the control mRNA level (Figure 2). The morphometrical characterization of SlTPL1 RNAi lines showed that two RNAi lines (line2, line3) had a similar fruit-set rate as the wildtype (WT), and line1 exhibited lower fruit-set rates than the WT (Figure 3A). There was no difference in fruit size (Figure 3B), fruit weight (Figure 3C), or seed number (Figure 3D) between the WT and the three RNAi lines, but the days from anthesis to breaker fruit stage were more in the RNAi lines than in the WT (Supplementary Figure 1C), indicating that fruit development was slower in the three RNAi lines than that in the WT.

**FIGURE 4:**
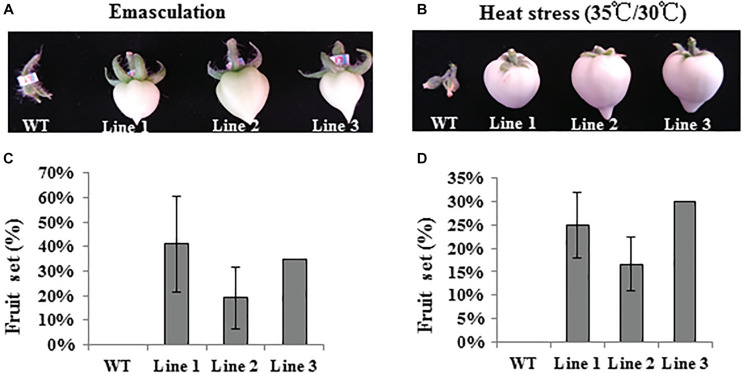
Fruit set analysis of WT and SlTPL1-RNAi plants after emasculation and under heat stress treatment. **(A)** Fruit photos of WT and SlTPL1-RNAi lines after emasculation treatment; **(B)** Fruit photos of WT and SlTPL1-RNAi lines after heat stress treatment; **(C)** Fruit set calculation of WT and SlTPL1-RNAi lines after emasculation treatment; **(D)** Fruit set calculation of WT and SlTPL1-RNAi lines after heat-stress treatment.

To further understand the function of *SlTPL1* on tomato fruit-set capacity, we emasculated WT and SlTPL1 RNAi flowers in the greenhouse to determine their fruit-set capacity (Figure 4A and Supplementary Figure 2A). Additionally, we cultured the WT and SlTPL1 RNAi lines in a growth chamber under 35/30°C day/night temperature regime as heat-stress treatment to check their fruit-set capacity (Figure 4B and Supplementary Figure 2C). The results showed that WT plants failed to set fruit, while the SlTPL1 RNAi lines set fruit at a rate that ranged from 19% to 43% after emasculation (Figure 4C). When the fruit turned red, we verified the seed number in them; no seeds were found in these fruits (Supplementary Figure 2B). Heat stress produced similar results with respect to fruit-set rate, with the WT losing the capacity to set fruit, whereas RNAi plants retained 16–30% of their fruit-set capacity (Figure 4D). No seeds were found in the seeded fruit of the progenies derived from the RNAi lines (Supplementary Figure 2D). Meanwhile, SlTPL1 was up-regulated by heat stress (Figure 1G).

### Measurement of Endogenous Hormone Levels in Emasculated Flower Buds of the WT and SlTPL1 RNAi Plants

As the hormone levels of ovaries are important for fruit set, we detected the endogenous hormone content in the ovaries of emasculated flowers of WT and SlTPL1-RNAi line1 plants. The results revealed that CKs (DZ, IP) levels in WT tomato plants were significantly lower than those in the emasculated flower buds of SlTPL1 RNAi plants at 2 days before anthesis (BA). The levels of active GAs, GA_1_ and GA_3_, showed no change. Conversely, ABA levels decreased (Table 1).

**TABLE 1:** Hormone levels in emasculated flower bud of WT and SlTPL1RNAi line1.

**Hormones**	**WT**	**TPL1RNAi**	**Type**	***t*-test**
ABA	81.37 ± 2.67	64.5 ± 2.404	down	**
DZ	238.33 ± 16.01	297 ± 2.82	up	*
IP	0.094 ± 0.008	0.146 ± 0.008	up	**
tZ	0.738 ± 0.03	0.696 ± 0.11	insig	*n*
ICAld	8.85 ± 0.44	7.3 ± 0.53	down	*
ME-IAA	0.434 ± 0.03	0.449 ± 0.04	insig	*n*
GA1	3.49 ± 0.37	3.83 ± 0.26	insig	*n*
GA3	3.79 ± 0.21	3.48 ± 0.57	insig	*n*

The data represent mean ± SE (*n* = 3). * indicate significant differences at *P* < 0.05. ** indicate significant differences at *P* < 0.01.

### Experimental Design for Transcriptomic Analysis of the Fruit-Set Process in Emasculated Flowers of SlTPL1-RNAi and WT Plants

As described above, SlTPL1-RNAi plants produced facultative parthenocarpic fruit. To further study the gene network involved in the regulation of facultative parthenocarpy, RNA-seq was performed on the emasculated fruit ovary at zero (E0) and 7 days (E7) after emasculation of fruit ovary. The complete experimental design included three parallel experiments. The first experiment was conducted to identify the genes whose expression is associated with the successful transition from ovary to fruit in SlTPL1-RNAi plants (SlTPL1RNAi E0 vs. SlTPL1RNAi E7). The second experiment was conducted to identify genes related to failure in the ovary-to-fruit transition in the WT (WT E0 vs. WT E7). Finally, the third experiment aimed to identify genes directly or indirectly regulated by SlTPL1 in the ovary tissues (E0, E7) that are common to WT and SlTPL1-RNAi plants (WT E0 vs. SlTPL1RNAi E0; WT E7 vs. SlTPL1RNAi E7). Both E0 and E7 included three biological replicates and generated 12 libraries. Among the 35,074 genes in the tomato genome, our RNA-seq data indicated that 93–95% of short clean reads were uniquely mapped to the tomato genome (*Solanum lycopersicum* ITAG4.0). The annotated gene numbers in the 12 libraries ranged from 22,509 to 23,970. Approximately, 594 novel transcripts were identified in the 12 libraries, each of which contained more than 530 novel genes (Supplementary Table 1).

**FIGURE 5:**
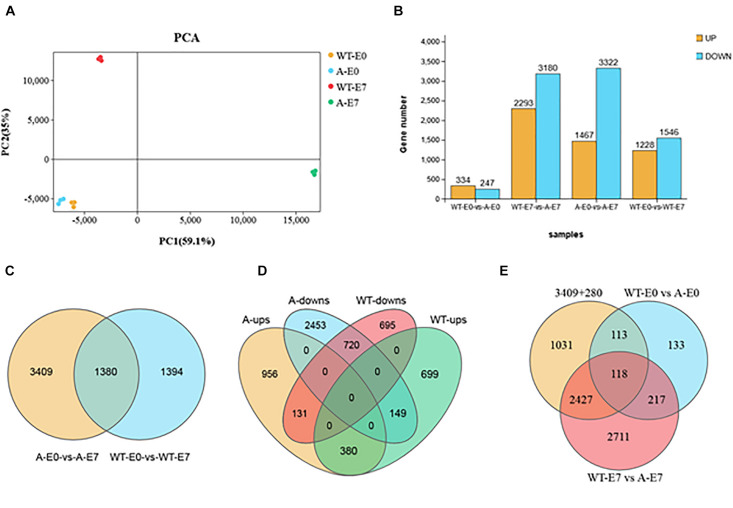
Differentially expressed genes (DEGs) analysis in WT and SlTPL1-RNAi plants after emasculation at E0 and E7 stages. **(A)** Principal component analysis (PCA) of the four group samples (WT-E0, yellow; A-E0, blue; WT-E7, red, and A-E7 green); the x-axis represents the first principal component and the y-axis represents the second; **(B)** Histograms showing the DEGs in WT and SlTPL1-RNAi plants after emasculation at E0 and E7 stages; **(C)** Venn diagrams showing the overlapping DEGs during fruit set process (E0 vs. E7) in ovaries of emasculated WT and SlTPL1-RNAi flowers; **(D)** Venn diagrams showing the overlapping DEGs of up-regulated and down-regulated DEGs during fruit set process (E0 vs. E7) in ovaries of WT and SlTPL1-RNAi flowers; **(E)** Venn diagrams showing the overlapping DEGs between 3689 specifically expressed genes during fruit set process and DEGs differently expression in WT and SlTPL1-RNAi ovaries at E0 and E7 stages. WT-E0: ovaries of wild type tomato at 0 day after emasculation; WT-E7: ovaries of wild type tomato at 7 days after emasculation; A-E0: ovaries of SlTPL1RNAi tomato at 0 day after emasculation; A-E7: ovaries of SlTPL1RNAi tomato at 7 days after emasculation.

To identify candidate genes that are vital for the ovary-to-fruit transition process, we performed a comprehensive analysis of gene expression related to the fruit set in WT failed ovaries and in SlTPL1-RNAi successful ovaries that completed the transition to fruit. In all, 4789 and 2774 differentially expressed genes (DEGs) in the fruit set were detected in SlTPL1-RNAi and WT plants, respectively (Figure 5B and Supplementary Table 2). Among them, 3409 and 1394 DEGs were specifically expressed in the transition of ovary to fruit in SlTPL1-RNAi and WT ovaries, respectively (Figure 5B and Supplementary Table 3). A total of 1380 DEGs were found to be common to both WT and SlTPL1-RNAi plants during the ovary-to-fruit transition (Figure 5C and Supplementary Table 3), and among common DEGs, 280 showed a reversed expression pattern (Figure 5D and Supplementary Table 3). Additionally, among the 3689 (3409 + 280) DEGs, 231 (113 + 118) were regulated by SlTPL1 in E0 ovaries (Figure 5E and Supplementary Table 4). Furthermore, of the 231 DEGs regulated by SlTPL1 in E0 ovaries, 118 showed SlTPL1-dependent regulation in both E0 and E7 ovaries (Figure 5E and Supplementary Table 4).

### Transcriptome Analysis of Failed and Successful Fruit Set in Ovaries of Emasculated Flowers of WT and SlTPL1-RNAi Plants

Principal component analysis (PCA) of the RNA-seq samples revealed highly repeatability of three replicates of each sample and huge differences among ovaries at the E7 stage between the wild type and SlTPL1RNAi (Figure 5A). Statistical analysis indicated that 1467 up-regulated DEGs and 3322 down-regulated DEGs were identified in the ovary-to-fruit transition in SlTPL1-RNAi plants (Figure 5B). In contrast, 1228 up-regulated and 1546 down-regulated DEGs were found in the ovary-to-fruit transition in the WT (Figure 5B). Using PlantGSEA, we subsequently performed a gene set enrichment analysis (GSEA) of DEGs identified in WT and SlTPL1-RNAi plants. Using *p* < 0.05 as significance threshold, nine and eleven Kyoto Encyclopedia of Genes and Genomes (KEGG) pathways were significantly enriched in WT and SlTPL1-RNAi plants, respectively (Tables 2, 3 and Supplementary Tables 5, 6). Seven of the nine pathways, namely, “Cutin, suberine and wax biosynthesis,” “Arachidonic acid metabolism,” “DNA replication,” “Nitrogen metabolism,” “Fructose and mannose metabolism,” “Brassinosteroid biosynthesis,” and “Glutathione metabolism,” were down-regulated in the WT (Table 2). By contrast, two of the nine pathways, “Butanoate metabolism,” and “Phenylpropanoid biosynthesis,” were up-regulated in the WT (Table 2 and Supplementary Table 5). Six pathways were up-regulated, while five were down-regulated in SlTPL1-RNAi plants. “Plant hormone signal transduction,” “Fructose and mannose metabolism,” and “RNA polymerase” were up-regulated in SlTPL1-RNAi plants, while “Oxidative phosphorylation,” “Galactose metabolism,” and “Nicotinate and nicotinamide metabolism” were down-regulated in SlTPL1-RNAi plants (Table 3 and Supplementary Table 6). “Fructose and mannose metabolism” and “Plant hormone signal transduction” showed a reversible trend in WT and SlTPL1-RNAi plants, indicating an important role of these pathway in successful ovary-to-fruit transition.

**TABLE 2:** Enriched gene sets in genes up-regulated and down-regulated in WT during fruit set process by PlantGSEA.

**Gene Set Name (No. Genes)**	**KEGG**	**No. Genes in Overlap**	***P*-value**	**Status**
Cutin, suberine and wax biosynthesis (40)	KO00073	5	0.014347	downs
Arachidonic acid metabolism (20)	KO00590	2	0.018803	
DNA replication (100)	KO03030	16	0.020833	
Nitrogen metabolism (36)	KO00910	6	0.025	
Fructose and mannose metabolism (83)	KO00051	2	0.041667	
Brassinosteroid biosynthesis (22)	KO00905	3	0.045524	
Glutathione metabolism (127)	KO00480	6	0.046917	
Butanoate metabolism (28)	KO00650	1	0.010571	ups
Phenylpropanoid biosynthesis (288)	KO00940	36	0.048504	

**TABLE 3:** Enriched gene sets in genes up-regulated and down-regulated in SlTPL1RNAi during fruit set process by PlantGSEA.

**Gene Set Name (No. Genes)**	**KEGG**	**No. Genes in Overlap**	***P*-value**	**Status**
Galactose metabolism (63)	KO00052	9	0.004860268	downs
Ascorbate and aldarate metabolism (67)	KO00053	12	0.007025761	
Nicotinate and nicotinamide metabolism (30)	KO00760	4	0.017069701	
Oxidative phosphorylation (385)	KO00190	11	0.030120483	
Linoleic acid metabolism (29)	KO00591	8	0.03303685	
Betalain biosynthesis (5)	KO00965	1	0.005791506	ups
RNA polymerase (111)	KO03020	4	0.006896552	
Phenylpropanoid biosynthesis (288)	KO00940	30	0.008376963	
Plant hormone signal transduction (410)	KO04075	19	0.01540154	
Fructose and mannose metabolism (83)	KO00051	10	0.024390243	
Homologous recombination (152)	KO03440	4	0.044982698	

### Transcriptome Analysis of Specific Gene Expression in the Ovary-to-Fruit Transition of Ovaries From Emasculated Flowers in SlTPL1-RNAi Plants

**FIGURE 6:**
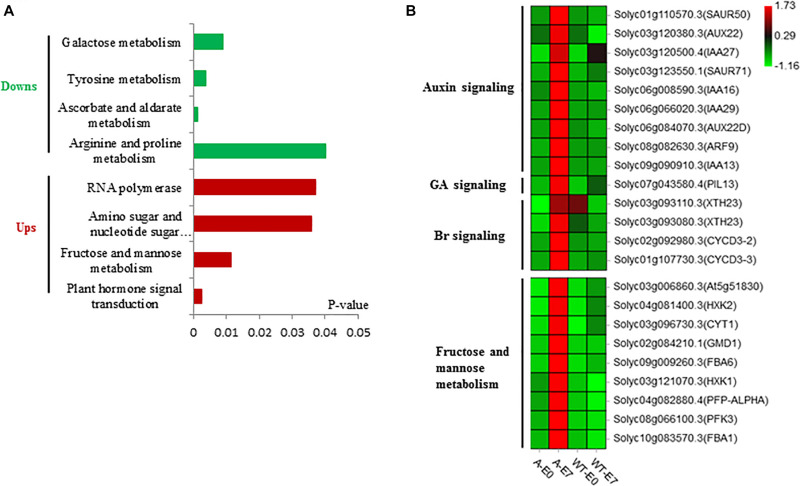
Functional analysis of DEGs specifically expressed in SlTPL1RNAi tomato ovaries. **(A)** Enriched gene sets of specifically up-regulated and down-regulated expressed genes in SlTPL1-RNAi plants during fruit set process as per PlantGSEA **(B)** heatmap of DEGs in gene sets of plant hormone signaling transduction and gene sets of fructose and mannose metabolism.

To identify candidate genes that are vital for the ovary-to-fruit transition process, we performed a comprehensive analysis of gene expression related to fruit set in WT ovaries that failed to set fruit and in SlTPL1-RNAi that successfully set fruit. In all, we found 3689 DEGs specifically related to successful fruit set in SlTPL1-RNAi plants, including 3409 DEGs specifically expressed in the SlTPL1-mediated fruit set process plus 280 DEGs common to WT and SlTPL1-RNAi plants, which were reversely expressed in SlTPL1-RNAi and WT during fruit set (Figure 5D and Supplementary Table 3). To gain further insight into the putative functions of these genes, all 3689 DEGs, of which 1087 were up-regulated and 2602 down-regulated, were collected for GSEA using PlantGSEA. Using *p* < 0.05 as significance threshold, four up-regulated and four down-regulated KEGG pathways were found to be significantly enriched in SlTPL1-RNAi plants (Figure 6A and Supplementary Table 7). “Fructose and mannose metabolism” and “Plant hormone signal transduction” were significantly up enriched in the SlTPL1-RNAi plants. The genes involved in auxin, GA, and Br signaling pathways were up-regulated in the successful fruit-set process of the SlTPL1-RNAi plants. In contrast, they were down-regulated or unchanged in the failed fruit-set process of WT plants (Figure 6B and Supplementary Table 7). Furthermore, genes involved in fructose and mannose metabolism were up-regulated in the fruit set of SlTPL1-RNAi plants but remained unchanged in the WT (Figure 6B).

### Transcriptome Analysis of SlTPL1-Dependent DEGs in the Ovary-to-Fruit Transition in Ovaries of Emasculated SlTPL1-RNAi Plants

We compared DEGs between WT and SlTPL1-RNAi in the ovary E0 and E7 in an attempt to reveal candidate genes responsive to *SlTPL1* down-regulation that might be function in fruit set in SlTPL1-RNAi plants. The results showed 581 DEGs in E0 ovaries between WT and SlTPL1-RNAi plants, among which 231 were also significantly and uniquely expressed in the SlTPL1-RNAi fruit-set process. Furthermore, among these 231 DEGs, 118 were differentially expressed in E0 and E7 samples between WT and SlTPL1-RNAi plants (Figure 5E and Supplementary Table 4).

**FIGURE 7:**
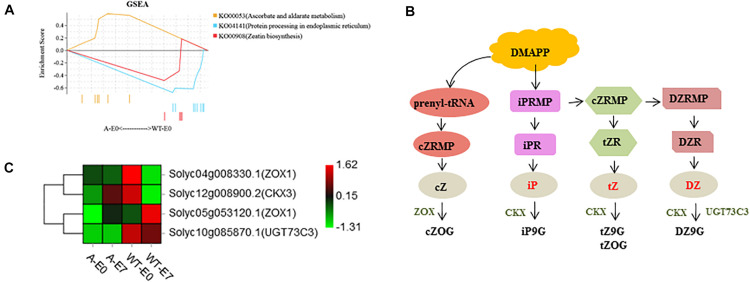
Functional analysis of DEGs specifically expressed in SlTPL1RNAi tomato ovaries at E0 stage. **(A)** Significantly enriched gene sets of DEGs in ovaries of emasculated flowers between WT and SlTPL1-RNAi plants at E0 stage; **(B–C)** Zeatin biosynthesis pathway and heatmap of DEGs in the gene set of Zeatin biosynthesis.

To gain further insight into the putative functions of these genes, all 581 DEGs were used for GSEA using PlantGSEA. Using *p* < 0.05 as significance threshold, three KEGG pathways, “Zeatin biosynthesis,” “Protein processing in endoplasmic reticulum,” and “Ascorbate and aldarate metabolism,” were significantly enriched (Figure 7A, Supplementary Figure 3, and Supplementary Table 8). DEGs involved in “Zeatin biosynthesis” were down-regulated in SlTPL1-RNAi after emasculation compared to that in WT (Figures 7B,C).

**FIGURE 8:**
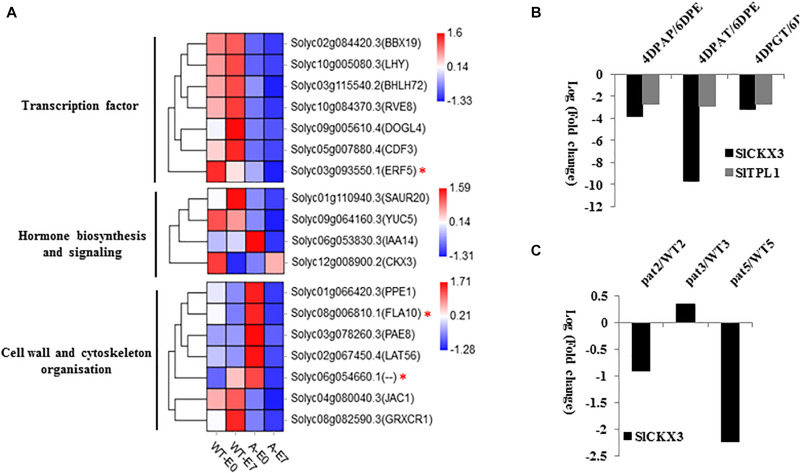
**(A)** Heatmap of DEGs in the gene set comprising 118 genes involved in hormone biosynthesis and signaling pathways, transcription factor, cell wall and cytoskeleton organization, and other genes involved in the fruit set process; **(B)** The expression data of *SlCKX3* and *SlTPL1* in tomato ovaries after artificial, auxin and GA triggered fruit set. 6DPE: 6 days post emasculation. 4DPAP: 4 days post artificial pollination. 4DPAT: 4 days post auxin treatment. 4DPGT: 4 days post gibberellin treatment. **(C)** The expression data of *SlCKX3* in pat ovaries. pat2: pat ovaries at 2 days before anthesis stage; pat3: pat ovaries at 0 days post anthesis stage. pat5: pat ovaries at 2 days post anthesis stage. WT2: wild type ovaries at 2 days before anthesis stage; pat3: wild type ovaries at 0 days post anthesis stage. WT5: wild type ovaries 2 days post anthesis stage.

To reduce the scope of SlTPL1-mediated gene regulation involved in facultative parthenocarpy, we performed functional categorization of the 118 DEGs in E0 and E7 samples between WT and SlTPL1-RNAi plants. Among the 118 DEGs, 18 were involved in cell wall organization, transcription factor, and hormone actions (Figure 8); four were found to be involved in phytohormone pathways: solyc06g053830.3.1 (IAA14), solyc01g110940.3.1 (SAUR20), solyc09g064160.3.1 (YUCCA), and solyc12g008900, (CKX3) (Figure 8). YUCCA is an enzyme involved in auxin biosynthesis. According to our RNA-seq data, YUCCA was down-regulated in the SlTPL1-RNAi samples. The expression of genes involved in hormone signaling also changed, such as the auxin-signaling component SAUR and IAA. CKX3 is related to CK metabolism, and its expression level was reduced in the E0 ovary of SlTPL1-RNAi plants. Based on the public RNA-seq data of tomato oaries involved in auxin, GA and artificial fruit set process (Ruiu et al., 2015; Tang et al., 2015), we found that SlTPL1 and SlCKX3 were both down-regulated in the auxin, GA and artificial triggered fruit set ovaries (Figure 8B). Meanwhile, we also found SlCKX3 was down-regulated in the pat ovaries (Figure 8C). All the genes involved in cell wall organization were induced in SlTPL1-RNAi plants. Five kinds of transcription factors were found: BBX-DBB, MYB, DOG, ERF, bHLH, and DOF (Figure 8). All these transcription factors were down-regulated in SlTPL1-RNAi plants. As previously reported, SlTPL1 interacted with most Aux/IAAs. We detected AuxRE in the promoters (3000 bp upstream of ATG) of the above 18 genes. The results showed that one ethylene-signaling pathway transcription factor ERF (solyc03g093550.1) and two genes (solyc08g006810.1 and solyc06g054660.1) involved in cell wall organization contained the auxin response element in their promoters (Figure 8), suggesting a possible network downstream of the auxin-signaling pathway-related genes.

### Validation of RNA-Seq Data by qRT-PCR

We amplified 13 genes by qRT-PCR using specific primers to confirm the accuracy and reproducibility of RNA-seq expression profiles. This results revealed that all 13 genes displayed the same trend, and the Pearson correlation coefficient between RNA-seq and qRT-PCR data was 0.92 (*P* < 0.0001), indicating that the RNA-seq was reliable (Supplementary Figure 4).

## Discussion

### SlTPL1 May Form a Complex With IAA and ARF to Repress Gene Transcription in Facultative Parthenocarpic Fruit Formation

The TPL/TPR family of co-repressors functions as a central regulatory hub regulating all nine phytohormone pathways and controlling plant development, including meristem maintenance, fruit ripening, and anthocyanin accumulation (Gallavotti et al., 2010; Fukazawa et al., 2014; Hao et al., 2014; Qu et al., 2019; Hu et al., 2020). As TPL proteins lack DNA-binding activity, they are incorporated into transcription complexes by interacting with transcription factors to repress gene expression in various processes (Causier et al., 2012, 2014). Previously, we showed that SlTPL proteins interact with most of the Aux/IAA proteins, implying an important role in auxin signaling (Hao et al., 2014). Here, auxin signaling was induced in SlTPL1-RNAi ovaries. Meanwhile, *SlTPL1* exhibited a pollination-dependent expression pattern, i.e., the expression level increased after pollination, implying an important role in the fruit-set process. *SlARF7*, whose down-regulation resulted in parthenocarpic fruit, showed a reverse expression pattern with *SlTPL1*. SlARF7 showed a sharp increase at the anthesis stage, and then decreased after pollination (de Jong et al., 2009b, 2011). It has been reported that ARF7 and IAA9 interact with each other, and both down-regulation of IAA9 and ARF7 additively affected the parthenocarpic fruit formation in tomato (Hu et al., 2018). Meanwhile, other ARF activators such as ARF5, ARF8b were also down-regulated in the ARF7 RNAi ovaries (Hu et al., 2018). Extensive interactome studies revealed that most Aux/IAAs interact with the TPL proteins. Additionally, ARF activators also interact with most Aux/IAAs, whereas no interaction between the ARF activator and TPL proteins has been reported (Causier et al., 2012, 2014; Piya et al., 2014). In our previous study, SlTPL1 has been reported to interact with most Aux/IAAs, including IAA9, whose down-regulation resulted in facultative parthenocarpy (Wang et al., 2009; Mazzucato et al., 2015). There is no interaction between SlTPL1 and ARF activators ARF7, ARF8, and ARF5, while IAA9 could interact with all of these activators in tomato. The down-regulation or up-regulation of these ARFs activators resulted in parthenocarpic fruit formation in tomato (Goetz et al., 2007; de Jong et al., 2009b, 2011). All of these data indicated that these ARF activators may function together in the fruit set process. In the SlTPL1RNAi ovaries, the auxin signaling pathway component were up-regulated (Figure 6B), and SlTPL1 was down-regulated in the auxin triggered parthenocarpy ovaries (Figure 8B). Based on the public RNA-seq data of dissected tomato ovaries (Pattison et al., 2015), we found that IAA9, ARFs activators and SlTPL1 expressed in all the tomato tissues (Supplementary Figure 5). Together with the parthenocarpy phenotypes and protein-protein interaction result we believed they may function together, but how? In Arabidopsis, TPL1 forms a complex with IAA12 and ARF5 to suppress the expression of auxin-responsive genes in the absence of auxin during embryogenesis (Szemenyei et al., 2008). We assumed that SlTPL1 may form a complex with IAA9 and ARF activators to suppress the expression of auxin-responsive genes in the fruit set process. However, the exactly working mechanism warrants further research.

### Increased CK Levels May Account for Facultative Parthenocarpy in SlTPL1-RNAi Plants

**FIGURE 9:**
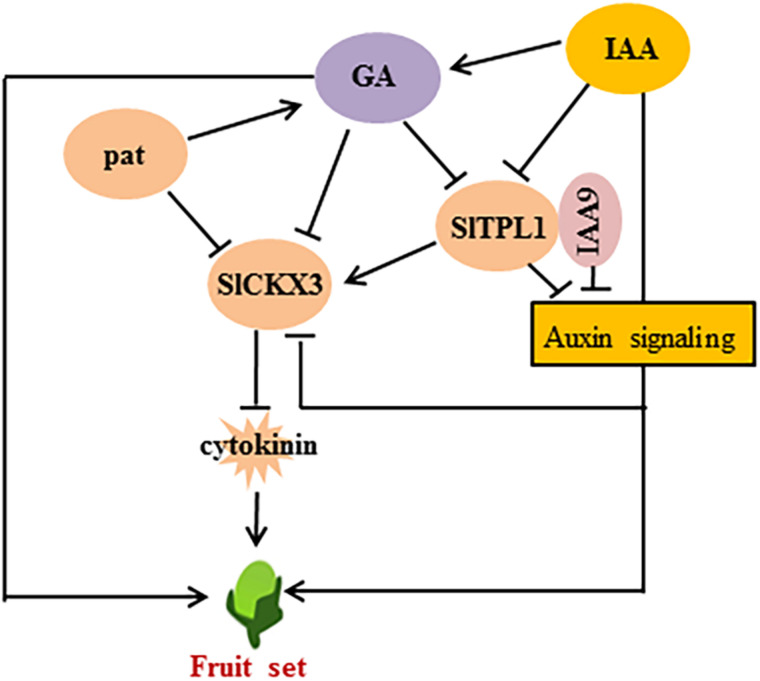
Hypothetical model showing the concerted action of S1TPL1 and SIIAA9 in the regulation of fruit set by modulating cytokinin metabolite related gene expression.

Hormone levels play an important role during ovary-to-fruit transition. IAAs and GAs are the main players in this process (Srivastava and Handa, 2005). Thus, exogenous IAA or GA treatment can trigger parthenocarpy without the need for pollination or fertilization (Mazzucato et al., 1999; de Jong et al., 2009a). Consistently, high IAA and GA levels are found in some parthenocarpic fruit mutants (Fos et al., 2000; Takisawa et al., 2018; Wang et al., 2020). The same situation was found in facultative parthenocarpic fruit; increased IAA and GA levels have been found in some facultative parthenocarpic mutants, such as *pat*, *pat2*, and *Auccisa* (Mazzucato et al., 1998; Fos et al., 2000; Molesini et al., 2009; Ribelles et al., 2019). Besides auxins and GAs, CKs and ABA are involved in the fruit-set process (Mariotti et al., 2011; Matsuo et al., 2012; Ding et al., 2013; Kai et al., 2019). Exogenous application of CKs induced parthenocarpy in tomato (Matsuo et al., 2012). The endogenous level of CKs has been shown to be directly correlated with fruit growth by promoting cell division (Ding et al., 2013). Recently, elevated CK levels were found in the *alq* mutant, which produced facultative parthenocarpic fruit under saline conditions. The *alq* ovaries exhibited higher pericarp thickness, which was associated with an increase in the number of cell layers. Meanwhile, CKs, which actively promote cell division, are significantly induced in the *alq* ovaries at anthesis. Thus, the increase in endogenous CKs is believed to be one of the factors determining early fruit set in *alq* (Ribelles et al., 2019). In our study, in addition to IAAs, GAs, and ABA, only the CK levels (DZ, IP) were higher in emasculated flower buds of SlTPL1-RNAi than in the WT. The genes involved in the cell cycle were up-regulated in SlTPL1-RNAi ovaries during the fruit-set process, while they were down-regulated in WT ovaries during fruit set. Meanwhile, the *CKX3*, involved in CK metabolism (Gasparis et al., 2019), was significantly down-regulated in emasculated flower buds, consistently with the increased CK levels observed in SlTPL1-RNAi plants. Based on the public RNA-seq data involved in tomato fruit set process, we found that *SlTPL1* and *SlCKX3* displayed similar expression pattern that they were both down-regulated in the artificial, auxin and GA triggered tomato fruit set ovaries. Meanwhile, *SlCKX3* was also down-regulated in the pat ovaries, indicating its important role in the parthenocarpy fruit formation. Coincidentally, there are TGA element, which is an auxin-responsive element and GA responsive element in the SlCKX3 promoter (Supplementary Table 9). All of these data indicated that *SlCKX3* is realted to the pathernocarpy fruit formation and it may function downstream of the auxin and GA. Therefore, we assumed that SlTPL1 likely regulates facultative parthenocarpy by down-regulating SlCKX3 expression, thereby allowing CK levels to increase (Figure 9). However, the specifics of this regulation warrants further research.

In facultative parthenocarpic mutants, the ABA levels are low in the ovaries (Ribelles et al., 2019). Previously, ABA was thought to be an additional player in the regulation of tomato fruit-set, together with other plant hormones (McAtee et al., 2013). Furthermore, ABA presumably inhibits ovary growth until fruit set, as ABA levels are high in mature ovaries but decrease after pollination (Kojima et al., 1993). However, application of ABA or ABA biosynthesis inhibitor fluridone neither inhibited nor increased the rate of fruit-set. Recently, new insights were gained into the role of ABA in the fruit-set process. Kai showed that overexpression or down-regulation of the ABA biosynthesis gene *SlNCED1* induced and reduced ABA levels in tomato anthers, resulting in poor pollen germination and pollen activity, thus, leading to poor fruit-set capacity (Kai et al., 2019). In our study, ABA levels were low in SlTPL1-RNAi ovaries, which may account for the low fruit-set rate observed under normal conditions.

### Other Hormones May Also Be Regulated by SlTPL1 During Fruit Set

Transcriptional co-repressors participates in GA signaling by interacting with GAF1 (Indeterminate domain 1 IDD1), which also interacts with DELLA to activate gene expression. GA converts the GAF1 complex from a transcriptional activator into a repressor via the degradation of DELLAs (Fukazawa et al., 2014). In our study, we found that the GA signaling component PIL3 was induced in SlTPL1-RNAi ovaries, but they were not dependent on SlTPL1 down-regulation. Meanwhile, the screening of Y2H library results revealed that SlTPL1 interacts with protein IDD (data not shown), which belongs to the indeterminate domain 1 protein family, indicating that SlTPL1 regulates facultative parthenocarpy by mediating GA signaling pathways.

The gaseous hormone ethylene, which plays an important role in fruit ripening, has been shown to suppress the initiation of fruit set by down-regulating GA accumulation (Shinozaki et al., 2015). The ethylene-insensitive-Sletr11 mutation produced parthenocarpic fruit, and fruit-set was effectively inhibited by PAC treatment. Sletr1–1 parthenocarpic fruits did not exhibit increased auxin accumulation but rather had increased levels of bioactive GAs, indicating that ethylene functions downstream of auxin but upstream of GAs (Shinozaki et al., 2015). In our study, the ethylene response factor 5, whose promoter contains an auxin-responsive element, was down-regulated in SlTPL1-RNAi plants at both E0 and E7 stages, indicating a possible role of ERF5 in connecting auxin-signaling and ethylene-signaling pathways in the fruit-set process. Further experiments are needed to confirm this finding.

The phenotypes of SlTPL1-RNAi lines along with the RNA-seq and protein–protein interaction data previously reported, implying that SlTPL1 together with IAA9 are instrumental in the regulation of fruit set by participating in the auxin-signaling pathway. The interaction between SlTPL1 and IAA9 seems to affect CK levels, thus, leading to ovary growth and promoting fruit set without pollination, which ultimately results in facultative parthenocarpic fruit formation. Overall, the outcome of this study adds to our understanding of the molecular factors involved in facultative parthenocarpy and provides potential targets for breeding strategies aimed at controlling this important trait.

## Data Availability Statement

The original contributions presented in the study are publicly available. This data can be found here: China National Center for bioinformation (https://bigd.big.ac.cn/gsa/), accession number CRA003992 (bigd.big.ac.cn/search/?dbId=gsa&q=CRA003992).

## Author Contributions

MH, SS, YL, ZP, MC, DC, and CW performed the experiments. YH participated in the design of the study and wrote the manuscript. YH, MZ, LC, and XZ analyzed the data. GH, BH, MZ, SS, RC, MB, and XZ assisted in revising the manuscript. All authors have read and approved the final version of the manuscript.

## Conflict of Interest

The authors declare that the research was conducted in the absence of any commercial or financial relationships that could be construed as a potential conflict of interest.

## Footnotes

1 http://www.Metware.cn/

2 https://sciex.com.cn/

3 www.omicshare.com/tools

## Supplementary Material

Supplementary Figure 1 Phenotyping of SlTPL1-RNAi tomato plants. Characterization of TSS content, Hue angle, fruit development, and ethylene production in SlTPL1-RNAi plants.

Supplementary Figure 2 Photographs of WT and SlTPL1-RNAi flowers after emasculation and under heat stress conditions. **(A)** Photos of emasculated flowers ovaries after 20 days emasculation; **(B)** Red fruits developed from ovaries of emasculated flowers of SlTPL1-RNAi lines. **(C)** Photos of plants cultured under heat stress. **(D)** Red fruits developed from ovaries of SlTPL1-RNAi lines under heat stress.

Supplementary Figure 3 Significantly enriched gene sets of DEGs in ovaries of emasculated flowers between WT and SlTPL1-RNAi plants at E0 stage.

Supplementary Figure 4 Validation of the expression data from RNA-seq assay by quantitative real-time PCR (qRT-PCR). **(A)** Relative expression levels of eight genes determined by qRT-PCR and FPKM obtained by RNA-seq. Results for each gene are based on three biological and three technical replicates. Error bars indicate standard error. **(B)** Pearson’s correlation between RNA-seq data and qRT-PCR data is reliable, with *R*^2^ > 0.8 as significance threshold. The values were row Z-score of RPKM and qRT-PCR.

Supplementary Figure 5 Heatmap of SlARFs activators, SlTPL1 and SlIAA9 in the dissections of tomato ovaries at anthesis and pollinated stages.
