# Supplementary material for: SlTPL1 Silencing Induces Facultative Parthenocarpy in Tomato
Source: Front Plant Sci. 2021 May 20;12:672232. doi: 10.3389/fpls.2021.672232 (PMC8174789; doi:10.3389/fpls.2021.672232)
Supplement: Supplementary Figure 1 — Phenotyping of SlTPL1-RNAi tomato plants. Characterization of TSS content, Hue angle, fruit development, and ethylene production in SlTPL1-RNAi plants. [file Data_Sheet_1.zip › Supplementary Table S1.docx]

Table S1. Summary statistics of RNA-seq data of the 12 libraries mapped to the tomato reference genome (*Solanum lycopersicum* ITAG4.0).

| Stage | WT-E0 | | | SlTPL1 RNAi-E0 | | | WT-E7 | | | SlTPL1 RNAi-E7 | | |
| --- | --- | --- | --- | --- | --- | --- | --- | --- | --- | --- | --- | --- |
| Library | WT-E0-1 | WT-E0-2 | WT-E0-3 | A-E0-1 | A-E0-2 | A-E0-3 | WT-E7-1 | WT-E7-2 | WT-E7-3 | A-E7-1 | A-E7-2 | A-E7-3 |
| Clean reads | 45629918 | 63038086 | 79895658 | 85284252 | 56886372 | 41310596 | 55274734 | 70235918 | 54355248 | 71633430 | 53670324 | 43642078 |
| High quality clean reads (%) | 45577884 (99.89%) | 62984162 (99.91%) | 79831948 (99.92%) | 85212646 (99.92%) | 56843118 (99.92%) | 41263408 (99.89%) | 55213970 (99.89%) | 70158164 (99.89%) | 54284652 (99.87%) | 71558150 (99.89%) | 53624360 (99.91%) | 43585530 (99.87%) |
| Removed rRNA reads (%) | 43467204 ( 95.37% ) | 60390020 ( 95.88% ) | 76606538 ( 95.96% ) | 82003930 ( 96.23% ) | 54120960 ( 95.21% ) | 39793078 ( 96.44% ) | 52659910 ( 95.37% ) | 67720176 ( 96.53% ) | 51700148 ( 95.24% ) | 69246336 ( 96.77% ) | 52075156 ( 97.11% ) | 42383294 ( 97.24% ) |
| Mapped reads(%) | 42368315 (97.47%) | 59031719 (97.75%) | 74639888 (97.43%) | 80207191 (97.81%) | 53101872 (98.12%) | 38819920 (97.55%) | 51604028 (97.99%) | 66010669 (97.48%) | 50559857 (97.79%) | 67490849 (97.46%) | 50994828 (97.93%) | 41249680 (97.33%) |
| Unique mapped reads (%) | 40795382 (93.85%) | 56690934 (93.87%) | 71675716 (93.56%) | 77167516 (94.10%) | 51142398 (94.50%) | 37389271 (93.96%) | 49854533 (94.67%) | 63579712 (93.89%) | 48665345 (94.13%) | 65434349 (94.50%) | 49489704 (95.04%) | 40063631 (94.53%) |
| Multiple mapped reads | 1572933 (3.62%) | 2340785 (3.88%) | 2964172 (3.87%) | 3039675 (3.71%) | 1959474 (3.62%) | 1430649 (3.60%) | 1749495 (3.32%) | 2430957 (3.59%) | 1894512 (3.66%) | 2056500 (2.97%) | 1505124 (2.89%) | 1186049 (2.80%) |
| All genes | 23200 (66.92%) | 23479 (67.72%) | 23781 (68.59%) | 23970 (69.14%) | 23524 (67.85%) | 23217 (66.97%) | 23324 (67.28%) | 23505 (67.80%) | 23385 (67.45%) | 22944 (66.18%) | 22572 (65.11%) | 22509 (64.93%) |
| Known genes (%) | 22649 (66.47%) | 22920 (67.26%) | 23224 (68.16%) | 23404 (68.68%) | 22961 (67.38%) | 22661 (66.50%) | 22778 (66.85%) | 22955 (67.37%) | 22831 (67.00%) | 22401 (65.74%) | 22042 (64.69%) | 21971 (64.48%) |
| Novel transcripts | 551 | 559 | 557 | 566 | 563 | 556 | 546 | 550 | 554 | 543 | 530 | 538 |
