# Supplementary material for: SlTPL1 Silencing Induces Facultative Parthenocarpy in Tomato
Source: Front Plant Sci. 2021 May 20;12:672232. doi: 10.3389/fpls.2021.672232 (PMC8174789; doi:10.3389/fpls.2021.672232)
Supplement: Supplementary Figure 1 — Phenotyping of SlTPL1-RNAi tomato plants. Characterization of TSS content, Hue angle, fruit development, and ethylene production in SlTPL1-RNAi plants. [file Data_Sheet_1.zip › Supplementary Table S9.docx]

3000bp promoters upstream of SlCKX3:

>SL2.50ch12:2216073..2213074

TCTCCATGGTTTAATATTAGCAAAGATTCGTTATCTTATTAGGATTGACAAGATAAAACCTAGTATTTATTTAATTTTTATAAAGATAACATAAATTTTTATTAGTGTTAAGAGCTAATTATATCATATCTTATTTATCAAATTACAAAAATTCCTTAAAATTTAAGAATTTCGAATATATCACTCAACGTCTTTGCTGATACAAGACATATCTATCCTGATAAATCGTTTAACGTCCCGATACATTACCTTTTGATACTAGGCACACATGCATGTCCGATACATCGTGTCCCGATACATCGCGATATAATTACATCGCTTTAGAATGATATATCTTATAGTAGAAGAGCTCGAGCGAGCTCGAACTGGACAATAGATAATTTTGTAATTTGTTTAAATTGTAGAAAAAAAATTAAAAATATGAAAAAAATAAATTATGTATTAATATAATTTTTCCTTAATTTTACTTACTCTTATTTGTGAAATTTAGTTTTTTAATTATCAAGAGGTAAATTTTATTTATCTATTTTTTAAAAGGAAGTAAAAGATAGGATCAATCGAAAGTGATGAAATCTACATTGAAATTGTCAAGAATATTGGTGGTAATTATAATTGCATGGAATTGACTTTTCATAATTCATATGTTACATCAAATACCTACAAATAAGATCTTACATTAAAGAAAAAAAAGATCTTAATTTGAATAAGGAAAATCATATTGATTTTTCCTTCAACTTTCCAATACATTGCCATTCTATGGTGTGTCAACTTTTTATTTGAAGATTCTTTTTATTTCCAAATCTTATTATTTTCTTATCTAATTAAATCAAAGATACTTGAAGATTTCATAAGATATTGAGTGTATAATTTTGGAAGTTACTTTTCTTATGTGCATACCTAGGCTTAGGGTGTGTTTGGTGAGAAGGAAACAATGTTTTTCATGAAATAAATTAATATAATAAGTAGAATTTATTTTTATTTTTTTAAAATGAAAAATGTTCAATTGTCATTGTCACAAATATTTTAATTGAATCAGAGAAAAAAACTTATGTCCAATGGCTATTCACAGTATTTTAATCAGAATTTATTTAATATTATGTGTTTTTTCAGTAAACTGATTTGGTGATAATGGAGAAAAAAATATCGTATTTTATAACACAATTTTTTCATCAATTCCTGTTGGAAAAAGTTTATGTTTCGAATAGTGAGCAAGTAAACCAAAAATATTAAAGATGTCTTTTGTCCACACACAAAGAATTATTAATAATTTCGTATATAATTTAGACAAATATTATAAATATACGGATATAGTGGTGATGGGTATGATGCAACATAATAGGAAGTATATAATCAAATATTAATAACACTCATAAAACTTTGAGAAATTATTTTTTAAAAATATTTTTCTCCGTATCAAGCACACCCATTTAATTATGATTATATGGTTCCTATATGTGAAACCAAGTAAATTCAAGAGGACATACTGATATAATTATTTACATTATAATAAATGCTAGATGCTAGATGTTTGGTATTATTATTCCACATAAGTATGAAGCACGTTTAACTTAAATAGATCCAAACAAAACAATAATAATAATAATAATAATAAATAAATAAATTATATCATTGGTTCAATTGATATACATGAAGTGGTTATATTAATTCAATAAATACATAATATATTTTCATGTATAACTTTTATCACAAACTATATACGCACAACAACAACAACAACAACAACAACAACAACAATAATAATAATAATAATAATAAATAAATAAATAAATAAATTATATCATTGGTTCAATTGATATACATGAAGTGGTTATATTAATTCAATAAATACATAATATATTTTCATGTATAACTTTTATCACAAACTATATACGCACAACAACAACAACAACAACAACAATAATAATAATAATAAATAAATAAATAAATTATATCATTGGTTCAATTGATATACATGAAGTGGTTATATTAATTCAATAAATACATAATATATTTTTATGTATAACTTTTATCACAAACTATAGTTTATAAGTACGCATTCTTAAGTTAATGTATAACATAATCATAATAAATTAGTATATTTTTGTATATACTAACCCTTTGTTCAAAAGAGGTAAAAATTTATTCGATATTTATATAAAGCCCATAAATATTTAGACATGTAATTACATTAGTTTTAACAATTAACCAAGGTTAATTAATTAAGATCACATGCAATTAATTCCCCTCATTAACTTAATTACTTTGCTATAATAAATTTAAATTATTTAAAACATCGAATATTGAAATAGTCACGAGAAAAATGCCCTATTCCTGTATATTCACAACAATTATCATGTCCTCTTATATTTCTATCTGGTCCTATTTATGATCGCATCTGTCTTATCCAAATGATAACGACTTCTATGACAAAAGACGCAAAGGAGGTTCAAGAACTTGCTACAGAAGACAACAAACTATCGATGAAAAGGTGGAAGATGAAACACGTTTTCTAATCCCTTTAAACGAGAAAGCTCCAAATAACTGCATACCGTTGATCAATAACATATACATCAATCTAAGATCTTGCTTACCGAATAATTAAAAAAAAATCTTCATTAATCATATAATGACAATACCATGAACAACATCAAAGAGGTGTTTATCCATCCCATGAGAAAATTGTGATGTTTTTTGGAACATAAAGGCAAAAGAGCAATAATCACAAAAATCTTTTGTCCTTTCATATCTTACACACACAATTTTCTACCCCCCACCCCCACCCCCACCCCACCCCCATGTGTAATATTATTTTTTCCAAGATCTTCTAAATCTTGTACTACCACTATATTAAAAAAATATTTTTTTTTACTTTTTTGCATGCAAATATTATTATTATTATATTATTTTCCTTCTCTATAAATACCCTCATATCACTCACTTTTCTCTTCACCAAAAATCACCTACTTCTAATAATTTCCTTTTTTTTTTCTTTCTTCTATT
