# Supplementary figures and images for: SlTPL1 Silencing Induces Facultative Parthenocarpy in Tomato
Source: Front Plant Sci. 2021 May 20;12:672232. doi: 10.3389/fpls.2021.672232 (PMC8174789; doi:10.3389/fpls.2021.672232)

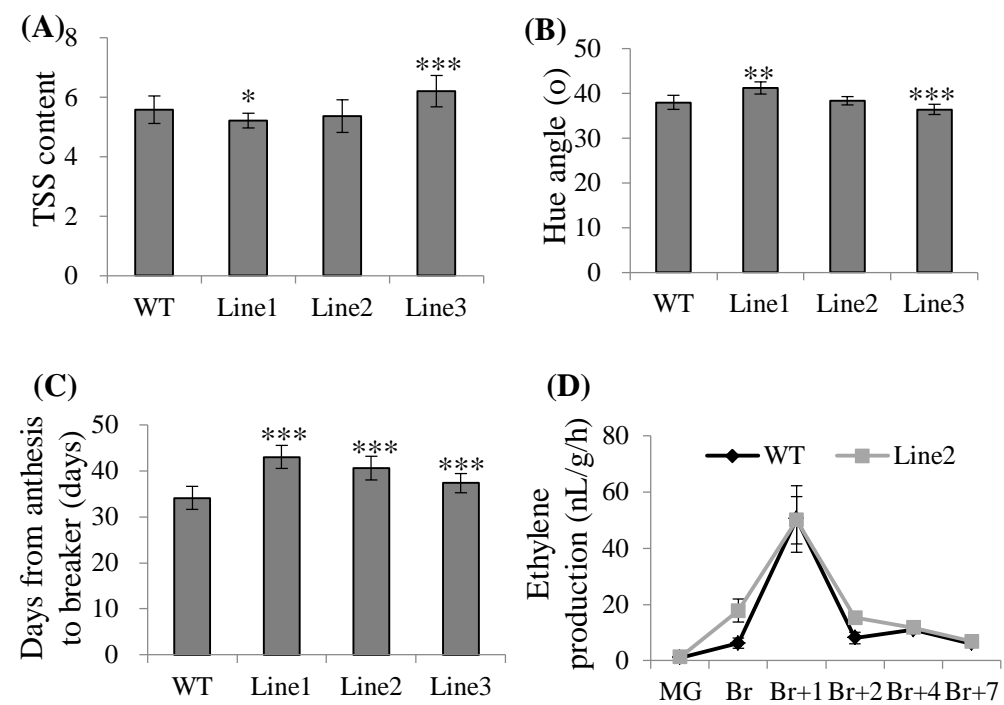

**FIGURE S1**

(A)

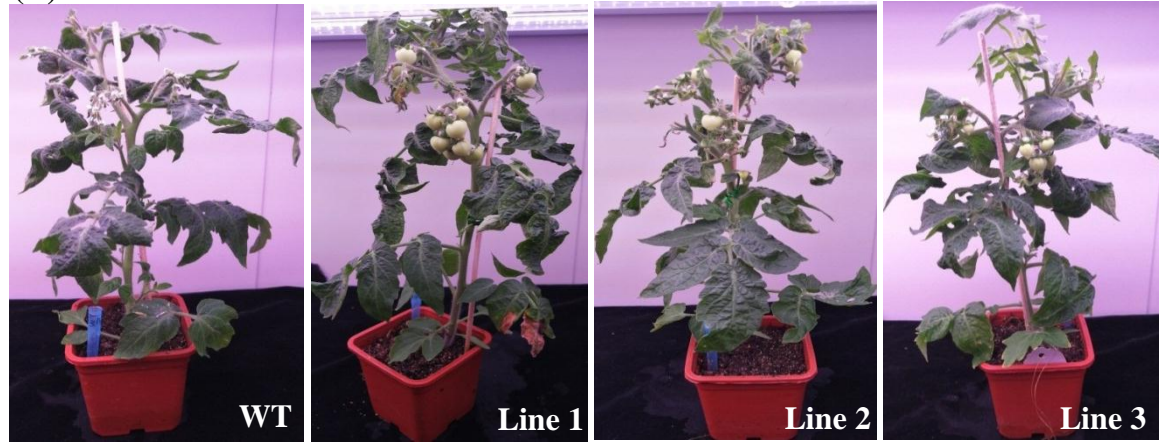

(B)

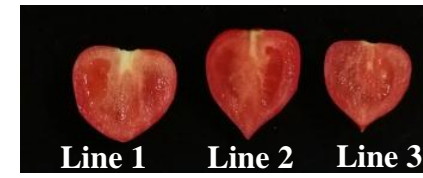

(C)

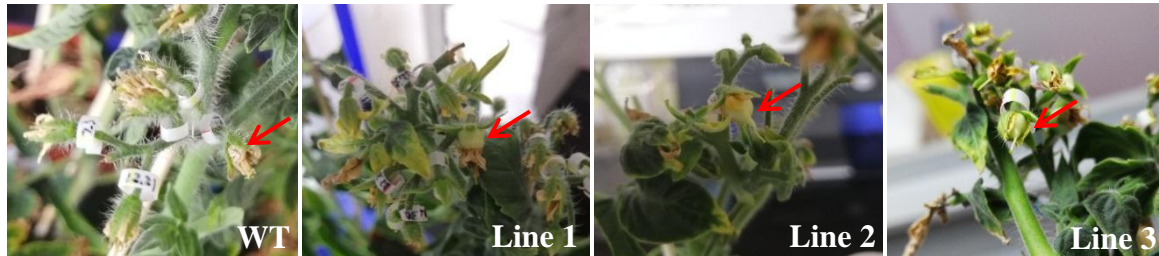

(D)

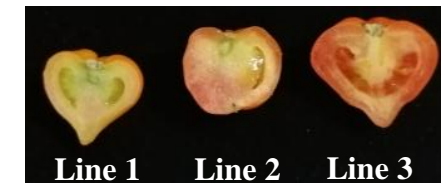

**FIGURE S2**

**(A)**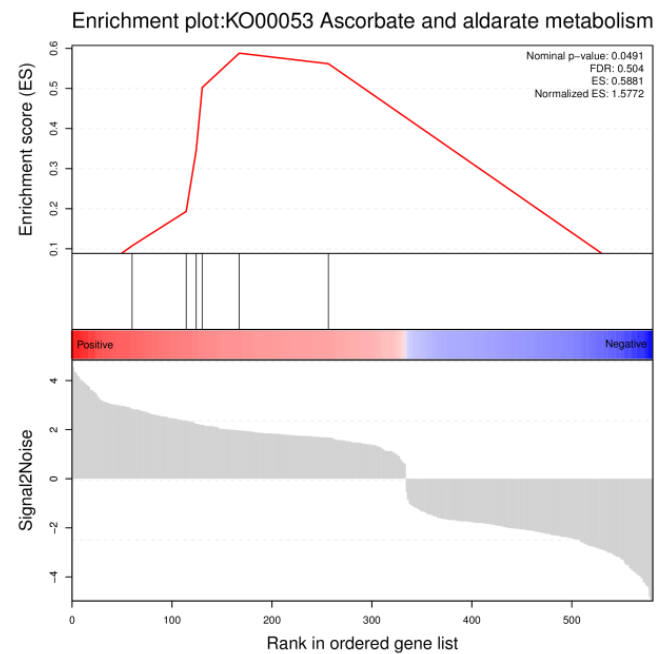**(B)**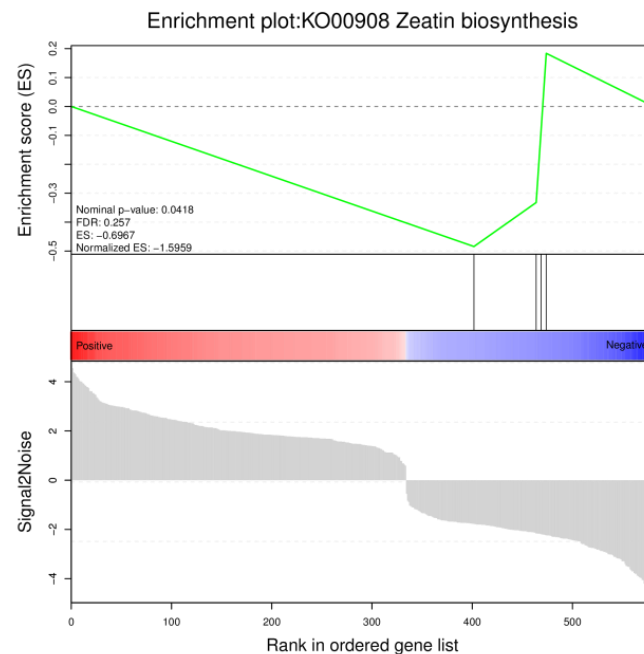**(C)**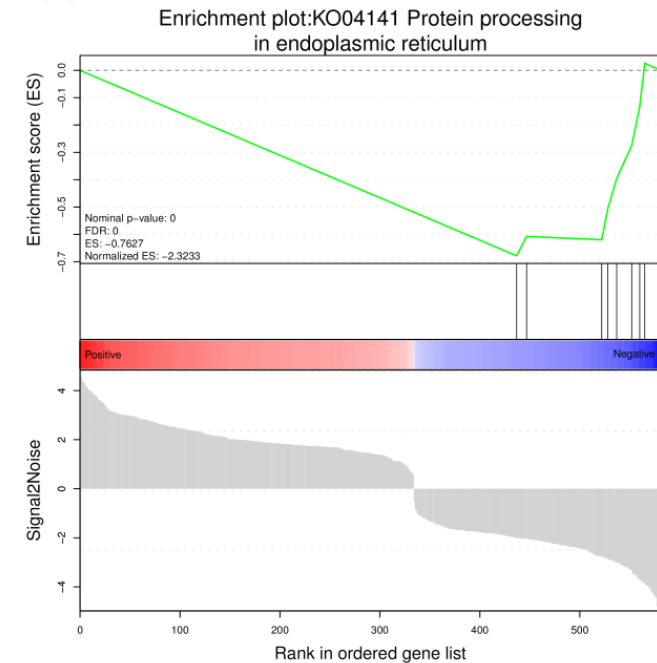**FIGURE S3**

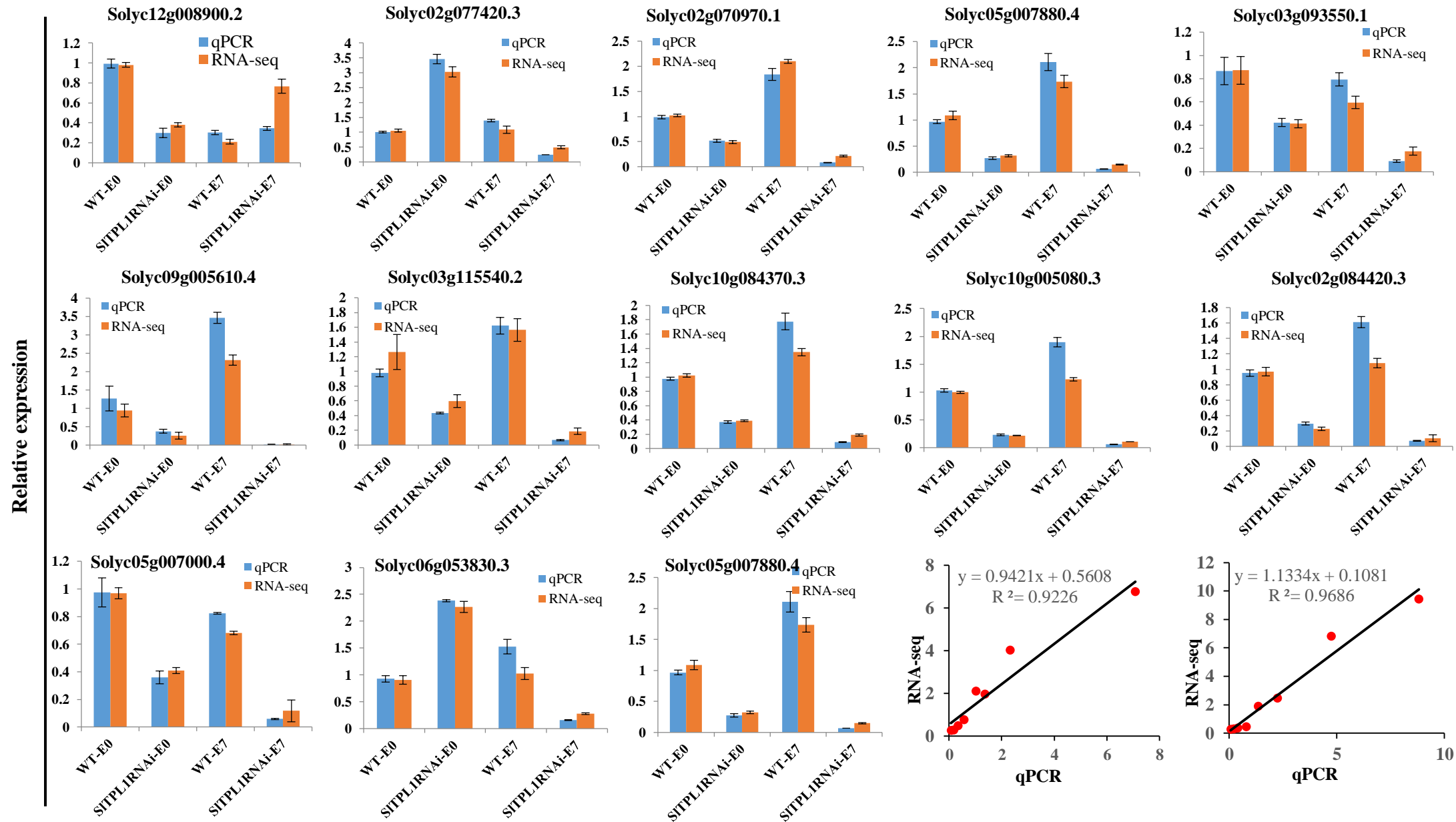

FIGURE S4

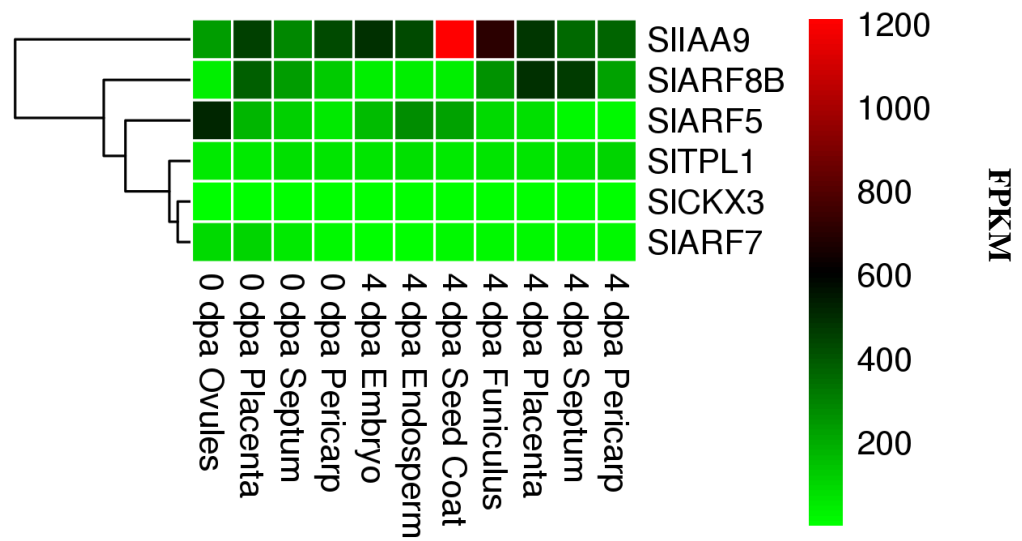

FIGURE S5

Supplement: Supplementary Figure 1 — Phenotyping of SlTPL1-RNAi tomato plants. Characterization of TSS content, Hue angle, fruit development, and ethylene production in SlTPL1-RNAi plants. [file Data_Sheet_1.zip › Supplementary figures.pdf]
